# Supplementary material for: Ethylene Induction of Non-Enzymatic Metabolic Antioxidants in Matricaria chamomilla
Source: Molecules. 2020 Dec 3;25(23):5720. doi: 10.3390/molecules25235720 (PMC7729440; doi:10.3390/molecules25235720)

Figure S1. Potential electrostatic maps (MEP) of selected quinic acid derivatives.

5-*O*-feruloyoquinic acid

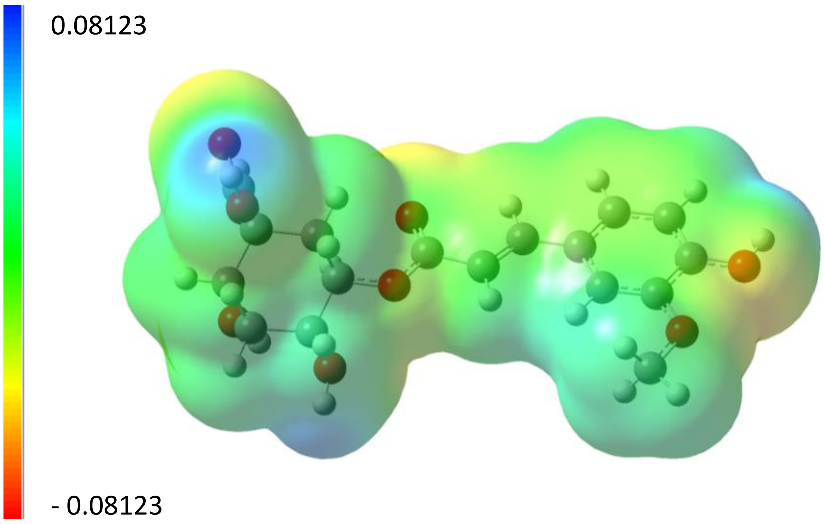

chlorogenic acid

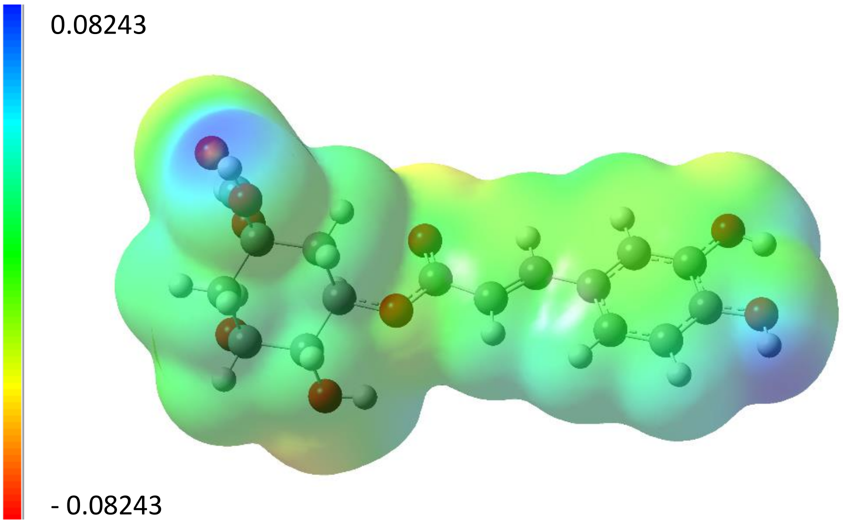

1,5-dicaffeoylquinic acid

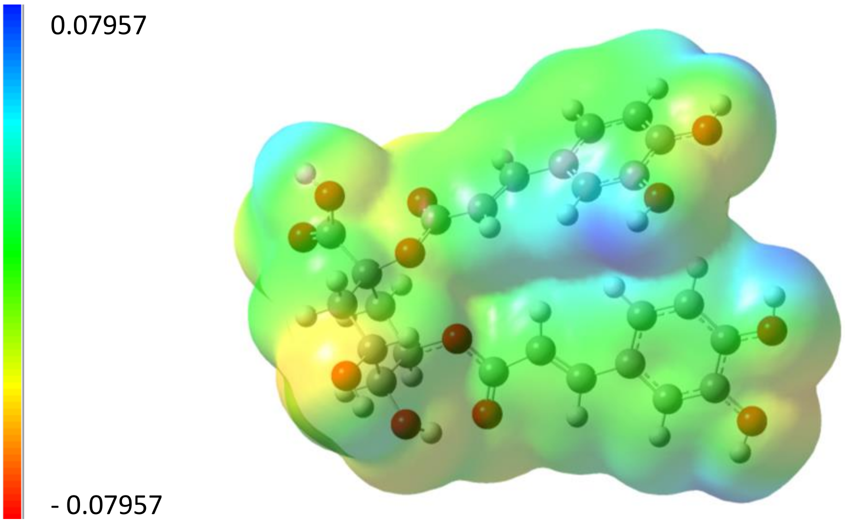

Supplement: Supplementary file 1 [file molecules-25-05720-s001.zip › Figure S1.pdf]
