# Supplementary material for: Ethylene Induction of Non-Enzymatic Metabolic Antioxidants in Matricaria chamomilla
Source: Molecules. 2020 Dec 3;25(23):5720. doi: 10.3390/molecules25235720 (PMC7729440; doi:10.3390/molecules25235720)

Figure S2. The frontier orbital HOMO and LUMO of selected quinic acid derivatives.

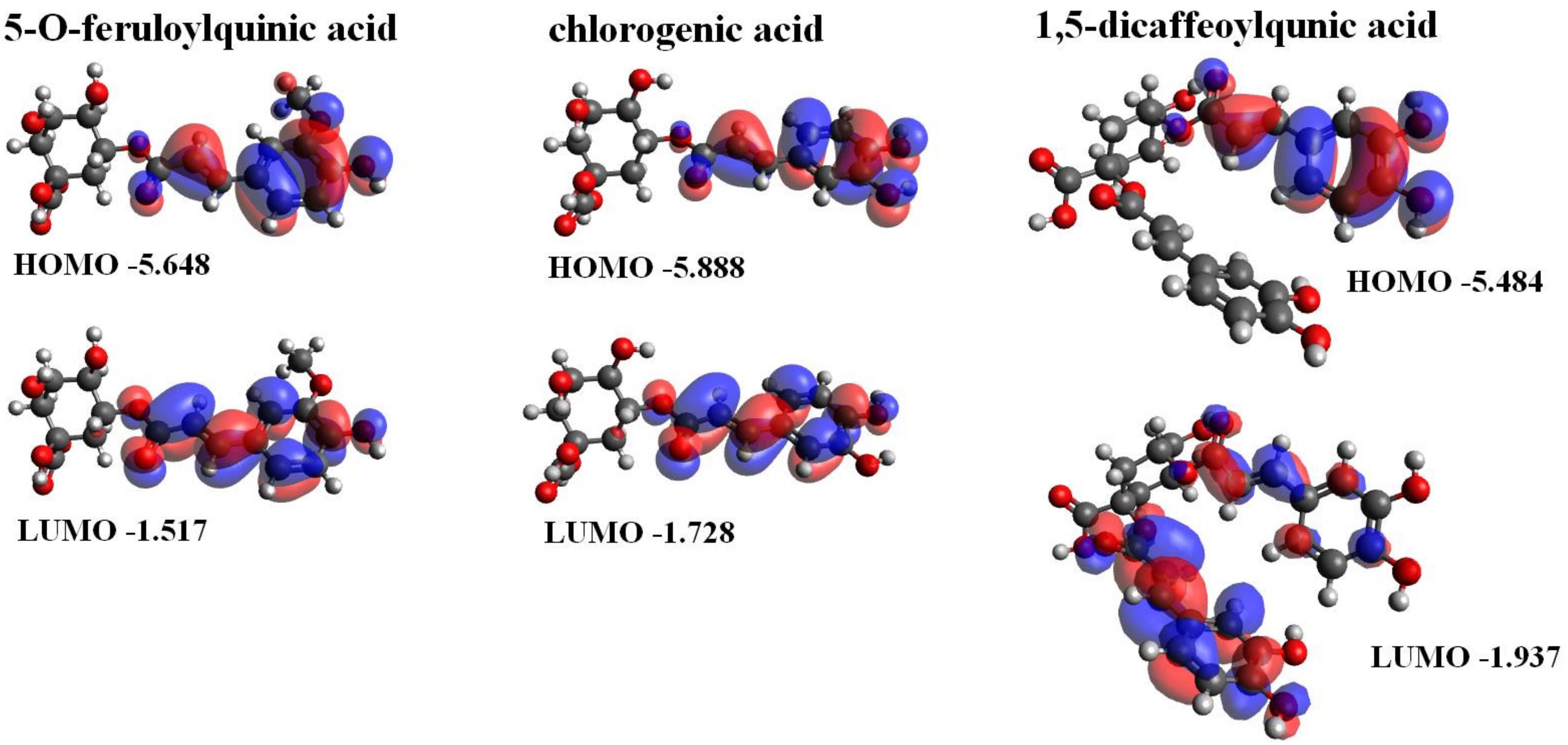

Supplement: Supplementary file 1 [file molecules-25-05720-s001.zip › Figure S2.pdf]
