# Supplementary material for: Ethylene Induction of Non-Enzymatic Metabolic Antioxidants in Matricaria chamomilla
Source: Molecules. 2020 Dec 3;25(23):5720. doi: 10.3390/molecules25235720 (PMC7729440; doi:10.3390/molecules25235720)
Supplement: Supplementary file 1 [file molecules-25-05720-s001.zip › Table S1.pdf]

Table S1: The predicted molecular properties confined to the druglike properties (based on Lipinski's rule of five).

	Molinspiration server predictions		
	**chlorogenic acid**	**5-FQA**	**1,5-DCQA**
miLogP	-0.45	-0.14	1.42
TPSA	164.74	153.75	211.28
Mol. Wt	354.51	368.34	516.46
nON	9	9	12
nOHNH	6	5	7
nviolations	1	0	3
nrotb	5	6	9
		SwissTargetPrediction	
target protein			
Enzyme Aldose reductase	1.00	0.28	0.53
Enzyme Aldo-keto reductase family 1 member B10	1.00	0.12	0.35
Protease Matrix metalloproteinase 2	0.18	0.17	0.22

The molecular properties for the compounds are predicted at molinspiration server to define the compounds druglikness. miLogP - Octanol-water partition coefficient; TPSA- topological polar surface area (drug transport properties); Mol. Wt (g mol^{-1}) - molecular weight, nON - number of hydrogen bond acceptors; nOHHN - number of hydrogen bond donors; nviolations - number of Lipinski's rule of five parameters violations; nrotb - number of rotatable bonds (molecular flexibility)
